# Quantitative Clinical Diagnostic Analysis of Acetone in Human Blood by HPLC: A Metabolomic Search for Acetone as Indicator

**DOI:** 10.1155/2016/5176320

**Published:** 2016-05-19

**Authors:** Esin Akgul Kalkan, Mehtap Sahiner, Dilek Ulker Cakir, Duygu Alpaslan, Selehattin Yilmaz

**Affiliations:** ^1^Department of Forensic Medicine, Faculty of Medicine, Canakkale Onsekiz Mart University, Terzioglu Campus, 17020 Canakkale, Turkey; ^2^Department of Leather Engineering, Faculty of Engineering, Ege University, Bornova, 35100 İzmir, Turkey; ^3^Department of Clinical Biochemistry, Faculty of Medicine, Canakkale Onsekiz Mart University, Terzioglu Campus, 17020 Canakkale, Turkey; ^4^Department of Chemical Engineering, Faculty of Engineering, Yuzuncu Yil University, 65080 Van, Turkey; ^5^Department of Chemistry, Faculty of Sciences and Arts, Canakkale Onsekiz Mart University, Terzioglu Campus, 17020 Canakkale, Turkey

## Abstract

Using high-performance liquid chromatography (HPLC) and 2,4-dinitrophenylhydrazine (2,4-DNPH) as a derivatizing reagent, an analytical method was developed for the quantitative determination of acetone in human blood. The determination was carried out at 365 nm using an ultraviolet-visible (UV-Vis) diode array detector (DAD). For acetone as its 2,4-dinitrophenylhydrazone derivative, a good separation was achieved with a ThermoAcclaim C_18_ column (15 cm × 4.6 mm × 3 *μ*m) at retention time (*t*_R_) 12.10 min and flowrate of 1 mL min^−1^ using a (methanol/acetonitrile) water elution gradient. The methodology is simple, rapid, sensitive, and of low cost, exhibits good reproducibility, and allows the analysis of acetone in biological fluids. A calibration curve was obtained for acetone using its standard solutions in acetonitrile. Quantitative analysis of acetone in human blood was successfully carried out using this calibration graph. The applied method was validated in parameters of linearity, limit of detection and quantification, accuracy, and precision. We also present acetone as a useful tool for the HPLC-based metabolomic investigation of endogenous metabolism and quantitative clinical diagnostic analysis.

## 1. Introduction

Acetone is the simplest ketone compound. In general, acetone is not considered harmful, and the World Health Organization has not classified acetone as carcinogenic. However, its prolonged inhalation can not only cause irritation of the mucous membranes, headaches, confusion, and narcotic effects, but lead to coma as well [1–5].

For etiological reasons, acetonaemia is classified to be of endogenous and exogenous origin [6, 7]. Multiple toxicities and physiopathological conditions result in ketosis (acetonaemia particularly), including diabetes mellitus (DM), starvation coupled with physiologic stress, prolonged exercise, during pregnancy, and ethanol toxicity, other alcohol ingestions, drug toxicities, inborn errors of ketone metabolism, alcoholic ketoacidosis, delirium tremens, and hypothermia [6–9]. DM is a disease involving environmental and genetic factors. The main symptom of DM is a high blood glucose concentration depending on insulin deficiency. In this case the body cannot fully use glucose but could use fatty metabolism instead of glucose for energy [9]. DM, especially diabetes and autoimmune associated diseases, thyroid disease, and diseases that can accompany diabetes (hypertension, cardiovascular disease, cerebrovascular disease, and renal insufficiency) can cause pathological changes in most of the tissues, organs, and biological fluids depending on lipotoxicity and glucotoxicity.

A lot of medical, chemical, and medicolegal investigations have been carried out with the determination of acetone in blood and other biological fluids [2, 6, 7, 10]. Over the decades, several methods have been used for its determination in biological samples. In the beginning, colorimetric methods were developed and used for the determination of acetone in plasma [11–13]. These methods have common disadvantages such as the lack of specificity and detection limit. In recent decades, gas chromatographs equipped with flame ionization detectors or mass spectrometric detectors have been developed for determination of acetone concentrations in body fluids and in expired air [14–18]. Enzymatic methods are more specific but more complex and have long assay times and gas chromatographic methods, although widely used, are applied with difficulty as routine tests [2]. The determination of acetone in the blood is most important in clinical diagnostic laboratory studies. There are three ketone bodies, while the two main ketone bodies are acetoacetate (AcAc) and 3-b-hydroxybutyrate (3HB), the third ketone body; acetone (Ac) is found minimum level [9]. Ketone bodies are produced by the liver and used peripherally as an energy source when glucose is not readily available [9, 19]. Ketone bodies are three water-soluble compounds that are produced as by-products when fatty acids are broken down for energy in the liver and kidney [19]. Ketone bodies are produced from acetyl-CoA mainly in the mitochondrial matrix of hepatocytes when carbohydrates are so scarce that energy must be obtained from breaking down fatty acids [9, 20]. Also, acetone is produced by spontaneous decarboxylation of acetoacetate [6–9]. Acetone cannot be converted back to acetyl-CoA, so it is excreted in the urine or exhaled [21]. Recently, blood or urine testing kits have been used to test for the presence of acetone in clinical biochemistry laboratories. Acetone can also be quantified by sampling the human blood and testing by gas chromatography [22]. Brega et al. described a rapid and simple HPLC procedure that can be used for the routine measurement of acetone in biological fluids, such as plasma and urine. According to Fujii et al., it is proposed that liquid chromatography with fluorescence (LC-FL) seems to be useful for the determination of acetone in the saliva [23].

In this paper, we present a rapid and simple HPLC technique using 2,4-DNPH as a derivatizing reagent for quantitative determination and metabolomic research of acetone in biological fluid such as human blood.

## 2. Material and Methods

### 2.1. Reagents and Standards

2,4-DNPH (Sigma-Aldrich, 97%), acetone (Merck, 99.8%), acetonitrile (Sigma-Aldrich, 99.8%), and methanol (Merck, 99.8%) were used in this study. All other chemicals were of analytical reagent grade and used without further purification.

### 2.2. Instrumentation

For the chromatographic analysis, Thermo Scientific Dionex Ultimate 3000 HPLC with a ThermoAcclaim-C_18_ (15 cm × 4.6 mm × 3 *μ*m) column and UV-Vis DAD were used. The deionized water was 18.2 M*Ω*·cm (Millipore Direct-Q3 UV) and was used throughout the experiments. For the biochemical analyses, Cobas 6000 (Roche, Germany) autoanalyzer was used for blood glucose levels. Qualitative analysis of total ketones in urine was evaluated by Iris Iricel 2000 Analyzer (Icem Velocity).

### 2.3. Sample Collection and Procedure for the Determination of Acetone in Human Blood

In the first stage of our study, following a 12-hour fasting venous blood samples were taken from patients admitted to hospital of the Faculty of Medicine, Canakkale Onsekiz Mart University. The human blood and urine samples were directly collected into a tube (without a collection device) between 08:30 and 11:00 am. Clinical Biochemistry Laboratory blood and urine glucose tests were studied for routine biochemistry using a urine autoanalyzer. Test results were screened for hyperglycemia and ketonuria. The patients were divided into high blood glucose and urine ketone positive subjects (Group 1) and high blood glucose and urine ketone negative example subjects (Group 2). The patients with hyperglycemia were 8 females and 7 males (age: 21–87; *n* = 15), while 5 female and 2 male patients had positive urine ketones (age: 21–68, *n* = 7) and 5 male and 3 female patients had negative urine ketones (age: 55–87, *n* = 8). The blood glucose levels varied between 110 and 320 mg/dL in our patients (Table 1).

In the second stage of the study, to determine the probable positive acetone, its quantitative analysis was carried out in biological fluids using the HPLC technique. The blood samples were immediately prepared for HPLC analysis carried out within 8 hours after sample collection. A method for the determination of acetone in human blood by HPLC was developed. Plasma specimens were deproteinized with acetonitrile (1 : 1, v/v); 2,4-DNPH is added to the supernatant (filtered blood samples) and treated with acetonitrile (2 : 1, v/v) to prevent crystallization of the synthesized phenylhydrazone. An aliquot (20 microliters) of the reaction mixture was subjected to HPLC at ambient temperature using ThermoAcclaim-C_18_ (15 cm × 4.6 mm × 3 *μ*m) column and UV-Vis DAD with (methanol/acetonitrile) water as eluent at a flowrate of 1 mL min^−1^ and detection at 365 nm [2, 3]. The experimental procedures were conducted in accordance with the ethical standards of the Helsinki Declaration and approved by the Canakkale Onsekiz Mart University Human Research Ethics Committee. Written informed consent was obtained from all the subjects.

### 2.4. Acetone Labeling and HPLC Analysis

The quantitative analysis of acetone using HPLC was performed after labeling with 2,4-DNPH. The 2,4-dinitrophenylhydrazone standards were prepared by mixing A and B solutions: (A): 0.40 g of 2,4-DNPH dissolved in 2.00 mL of H_2_SO_4_ + 3.00 mL of H_2_O + 10.0 mL ethanol; (B): 0.50 g or 1.00 mL of the acetone standard dissolved in 20.0 mL ethanol. After this mixing, a precipitate was formed in each case, isolated through filtration, and dried in vacuum [3, 24, 25].

Acetone was added into its 2,4-DNPH derivatives by mixing 1.00 mL of a solution containing 200 mg/100 mL of 2,4-DNPH with 1.0 mL of H_3_PO_4_, and 4.00 mL of the human serum. After 2 h, a 40.0 *μ*L aliquot was withdrawn and analyzed by the HPLC technique [3, 24–27]. Chromatographic separation was achieved in a ThermoAcclaim-C_18_ (15 cm × 4.6 mm × 3 *μ*m) column at UV-Vis DAD detector (*λ*_max_ 365 nm). The injection volume was 20.0 *μ*L and the detection was performed at 365 nm. The following gradient was used: (methanol/acetonitrile) (8 : 2) water 60 : 40 (v/v). Elution was achieved at retention time (*t*_R_) 12.10 and flow-rate of 1 mL min^−1^.

### 2.5. Preparation of Calibration Standards

Standard calibration curve was prepared with acetone (0.5, 2.5, 5.0, 10, and 20 mmol L^−1^) and 40 *μ*L 2,4-DNPH in acetonitrile. Human samples were prepared by adding 40 *μ*L 2,4-DNPH and 500 *μ*L acetonitrile to 200 *μ*L human serum. The calibration curve was constructed by plotting the peak area of the 2,4-DNPH derivative of acetone (*y*) versus the concentration of acetone (*x*, mmol L^−1^) by linear regression (*n* = 4). The equation was found as *y* = 0.7361*x* + 0.0877 with a 0.9967, correlation coefficient (*R*).

### 2.6. Method Validation

The method proposed was validated as described in ICH guidelines in parameters of linearity, limit of detection and quantification, accuracy, and precision [28].

#### 2.6.1. Linearity

The linearity of the method was determined at four concentration levels ranging from 0.5 to 20 mmol L^−1^. The calibration curves were constructed by plotting the peak area of the 2,4-DNPH derivative of acetone (*y*) versus concentration of acetone (*x*). The slope, *y*-intercept, and correlation coefficient were calculated. To check the linearity, *F* test also was applied [28–33].

#### 2.6.2. Limit of Detection (LOD) and Limit of Quantification (LOQ)

The LOD was estimated using signal-to-noise ratio of 3 : 1 or (3 s/m) and LOQ as 10 : 1 or (10 s/m), at which accuracy and standard deviation were within 20% as per ICH [28, 32, 34, 35].

#### 2.6.3. Accuracy and Precision

Intraday accuracy and precision were performed for acetone at 5.0 mmol L^−1^ in replicate (*n* = 3). Interday accuracy and precision were achieved on three different days. Accuracy (expressed as recovery) and precision (expressed as relative standard deviation) should not deviate by ±15% of the nominal concentration [34].

## 3. Results and Discussion

### 3.1. The Chromatogram of 2,4-DNPH

**Figure 1:**
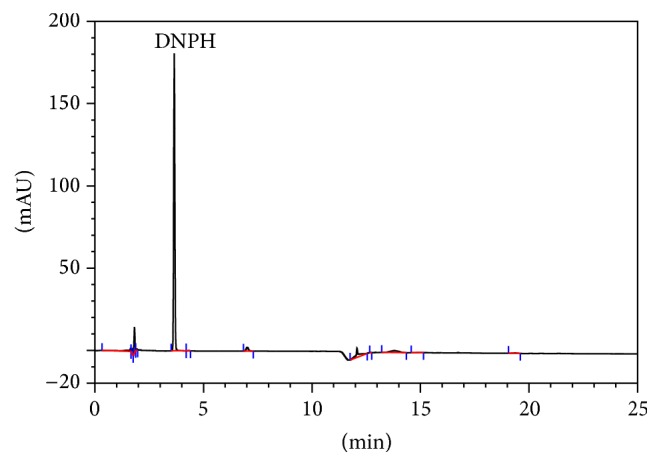
The chromatogram of 2,4-DNPH; for chromatographic conditions: see Section 2.

Elution and recovery problems were solved using HPLC with 2,4-DNPH as derivatizing reagent. The chromatogram of 2,4-DNPH is given in Figure 1. As can be seen from Figure 1, the retention time (*t*_R_) was obtained as 3.80 min.

### 3.2. Typical HPLC Chromatogram of Acetone as Its 2,4-DNPH Derivative

**Figure 2:**
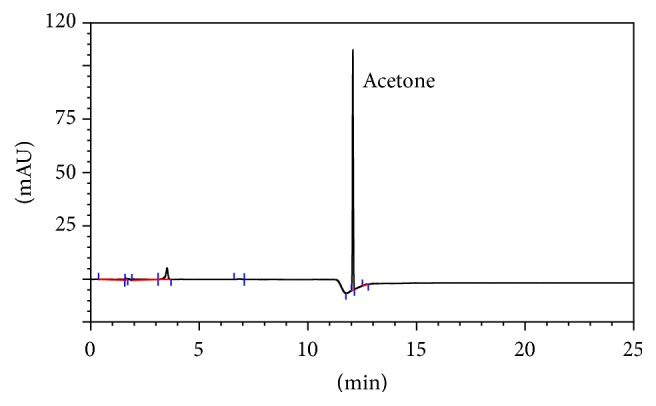
: Typical HPLC chromatogram of 0.681 mmol L^−1^ acetone as its 2,4-DNPH derivative; for chromatographic conditions: see Section 2.

The most efficient separation of acetone as its 2,4-DNPH was obtained with a ThermoAcclaim C_18_ column (15 cm × 4.6 mm × 3 *μ*m) at retention time (*t*_R_) 12.10 min and flowrate of 1 mL min^−1^ using a (methanol/acetonitrile) water elution gradient. No elution problem for acetone as its 2,4-dinitrophenylhydrazone derivative was observed. Typical HPLC chromatogram of 0.681 mmol L^−1^ acetone as its 2,4-DNPH derivative is given in Figure 2.

### 3.3. Determination of Acetone in Human Blood by HPLC

**Figure 3:**
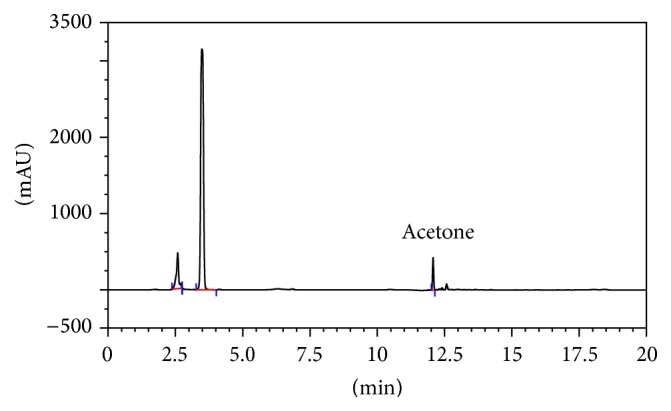
The HPLC chromatogram of 15 mmol L^−1^ acetone in human blood from first patient of Group 1; for chromatographic conditions: see Section 2.

The HPLC chromatogram of 15 mmol L^−1^ acetone in human blood in the first patient of Group 1 is given in Figure 3.

### 3.4. Quantitative Determination of Acetone in Patients

In our study, the patients were determined as high blood glucose and urine ketone positive subjects (Group 1) and high blood glucose and urine ketone negative example subjects (Group 2). Test results were screened for hyperglycemia and ketonuria. The blood glucose levels, given in Table 1, vary between 110 and 320 mg/dL in our patients, except for sample 7. Many patients in our study have DM disease, except samples numbered 4, 5, 11, 13, and 15 (Table 1).

### 3.5. Method Validation

#### 3.5.1. Linearity

The calibration plot of peak area against concentration was obtained linear in the range 0.5 to 20 mmol L^−1^. The regression equation and correlation coefficient were obtained as *y* = 0.7361*x* + 0.0877 with a 0.9967, correlation coefficient (*R*).

*F* test applied to accuracy of calibration curve. For calibration curve, *F*_critical_ value at 12 degrees of freedom (*p* = 0.05) is 2.20. Experimental *F* value is obtained as 1.26. So this value is smaller than 2.2, and calibration curve is linear. For the human plasma samples, *F*_critical_ value for calibration curve at 3 degrees of freedom (*p* = 0.05) is 3.18. Experimental value is obtained as 2.03. So, this value is smaller than *F*_critical_ value, and obtained values are appropriate.

#### 3.5.2. Limit of Detection and Quantification

The LOD and LOQ were found as 0.041 and 0.136 mmol L^−1^, respectively.

#### 3.5.3. Accuracy and Precision

Accuracy and precision results are summarized in Table 2. For intraday assay, the accuracy for acetone in human plasma samples expressed as recovery was found as 98%. For interday assay, the accuracy for acetone in human plasma samples expressed as recovery was found as 96%.

### 3.6. Mechanism of 2,4-DNPH to 2,4-Dinitrophenylhydrazone

*Brady's Test*. 2,4-DNPH can be used to qualitatively detect the carbonyl functionality of a ketone such as acetone or aldehyde functional group. A positive test is signaled by a yellow, orange, or red precipitate known as a dinitrophenylhydrazone. If the carbonyl compound is aromatic, then the precipitate will be red; if aliphatic, then the precipitate will have a more yellow color [36]. The reaction between 2,4-DNPH and a ketone such as acetone is shown below:

(1) RR′CO+C6H3NO22NHNH2⟶C6H3NO22NHNCRR′+H2O

**Scheme 1:**
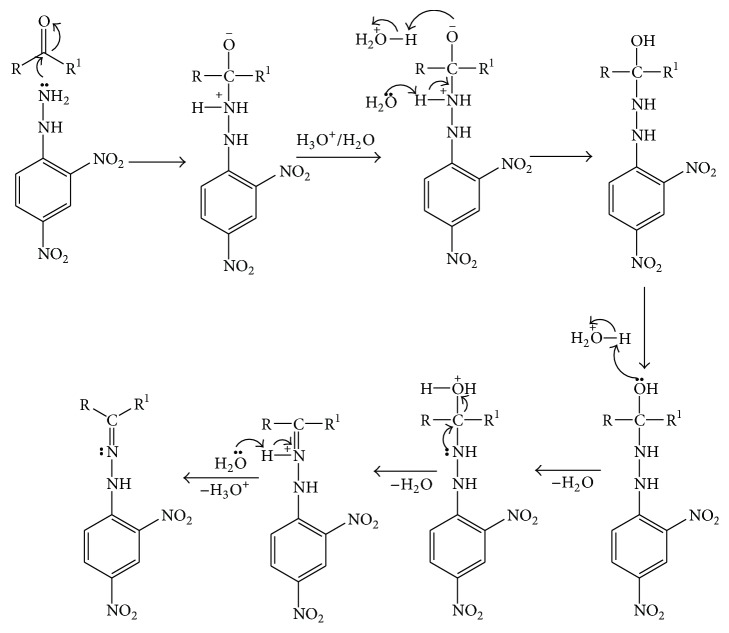
Mechanism of 2,4-dinitrophenylhydrazine to 2,4-dinitrophenylhydrazone.

This reaction can be described as a condensation reaction, with two molecules joining together with loss of water. It is also considered an addition-elimination reaction: nucleophilic addition of the -NH_2_ group to the C=O carbonyl group, followed by the removal of an H_2_O molecule. The mechanism of 2,4-DNPH to 2,4-dinitrophenylhydrazone is given in Scheme 1 [37].

2,4-DNPH does not react with other carbonyl-containing functional groups such as carboxylic acids, amides, and esters. For carboxylic acids, amides, and esters, there is resonance associated stability as a lone pair of electrons interacts with the p-orbital of the carbonyl carbon resulting in increased delocalization in the molecule. This stability would be lost by addition of a reagent to the carbonyl group. Hence, these compounds are more resistant to addition reactions. Also with carboxylic acids there is the effect of the compound acting as a base, leaving the resulting carboxylate negatively charged and hence unable to be attacked by this nucleophile [36, 37].

## 4. Conclusions

In this study, an analytical method was applied for the quantitative determination of acetone in human blood. The determination was carried out using a UV-Vis DAD detector with HPLC. In most of our patients' blood samples, high level of acetone has been determined. Higher levels of acetone have been measured in the patients who have high level of blood glucose and positive urine ketone (Group 1). The HPLC method based on the labeling of acetone with 2,4-DNPH seems to offer a rapid, low cost, sensitive, selective, and reproducible methodology for quantification of the acetone level in clinical samples such as human blood. The HPLC method described here overcomes many of the problems in the determination of acetone in biological fluids and the preanalytical errors. The volatile ketone is promptly stabilized by conversion into its DNPH derivative and rapidly determined without recourse to a solvent extraction step. The method uses very inexpensive reagents. As can be stated by Brega et al., the proposed HPLC method can therefore be used to great advantage over current gas chromatographic methods; it can be used in experiments requiring multiple samples and specific activity determination for the routine measurement of acetone in diabetic patients and in biological monitoring of exposed workers. The use of very small sample amounts makes this a favourable method in pediatrics [2].

We also presented acetone as a useful tool for the HPLC-based metabolomics investigation of endogenous metabolism and quantitative clinical diagnostic analysis. In the medical and medicolegal practice, the level of acetone could not be characteristic for a specific disease. However, the detection and early identification of acetone could be used as an initial indicator of detection of all these physiopathological conditions [7] and the biological monitoring test [2] and to determine further diagnostic management and timely treatment. Determination of acetone levels in blood may be a valid clinical approach in the symptomatic or/and nonsymptomatic cases in the literature for determining treatment strategy and controlling glycemic levels of patients. Consequently, the advanced studies which evaluate blood and urine ketone bodies level together should be performed in different physiopathological conditions including clinical and forensic toxicological studies.

## Figures and Tables

**Table 1:** Quantitative analysis data of acetone as its 2,4-dinitrophenylhydrazone derivative in human blood by HPLC.

Sample	Subject	Sex	Age	Blood glucose	Urine ketone	Acetone levels by HPLC (mean value ± sd.)^*∗*^
						Blood (mmol L^−1^)^*∗∗*^
*Group 1*						
1	Type 1 diabetes mellitus, Hashimoto disease, systemic lupus erythematosus	F	21	197	++	15.00 ± 1.00
2	Diabetes mellitus (DM), pneumonia	M	68	320	+	2.40 ± 0.20
3	DM, obesity, depression	F	33	196	+	3.86 ± 0.10
4	Acute pancreatitis	M	60	134	+	0.013 ± 0.001
5	Acute pancreatitis, obesity, hyperlipidemia	F	33	117	+	0.22 ± 0.01
6	DM	F	37	175	+	0.24 ± 0.01
7	Gestational DM	F	37	74	+++	17.27 ± 1.00

*Group 2*						
8	DM, hypothyroidemia	M	80	340	−	0.42 ± 0.01
9	DM, hypertension	F	82	248	−	3.20 ± 0.10
10	DM, hypertension, cerebrovascular disease, acute kidney failure	F	77	202	−	3.10 ± 0.10
11	Anemia, hypothyroidemia, hyperlipidemia	M	52	110	−	0.033 ± 0.01
12	DM, hypertension, hyperlipidemia	F	55	137	−	1.53 ± 0.01
13	Hypertension, congestive heart disease, chronic obstructive pulmonary disease, cerebrovascular disease	M	83	182	−	1.51 ± 0.01
14	DM, larynx carcinoma, hypothyroidemia	M	59	177	−	1.54 ± 0.01
15	Hepatic failure, hypertension, chronic kidney failure	M	87	111	−	2.13 ± 0.10

^*∗*^Number of repeated experiments, *n* = 4, and ^*∗∗*^reference value: 0.034–0.120 mmol L^−1^ with a mean value in plasma: 0.075 mmol L^−1^ (*n* = 20) [2].

**Table 2:** Intraday and interday precision and accuracy of the applied method.

Analyte	Period of analysis	Nominal concentration mmol L^−1^	Mean concentration found mmol L^−1^	Recovery%	RSD%	*n*
Acetone	Intraday	0.50	0.49 ± 0.01	98	2.04	3
	Interday	0.50	0.48 ± 0.01	96	2.10	3
